# Human Embryonic Stem Cell‐Derived Immunity‐And‐Matrix‐Regulatory Cells Attenuate Pulmonary Fibrosis via MMP1‐Mediated Collagen Degradation

**DOI:** 10.1111/cpr.70267

**Published:** 2026-08-05

**Authors:** Zhongwen Li, Dingyun Song, Faguo Sun, Licheng Song, Bin An, Tingting Gao, Jingjing Liu, Kan Zhang, Kaidi Liu, Kaiwei Wu, Yajing Li, Yi Yang, Yaru Liu, Ruofan Su, Zai Wang, Tiemei Zhao, Huaiyong Chen, Li Xiao, Liang Peng, Liu Wang, Wei Li, Lixin Xie, Jie Hao, Huaping Dai, Jun Wu

**Affiliations:** ^1^ State Key Laboratory of Organ Regeneration and Reconstruction Institute of Zoology, Chinese Academy of Sciences Beijing China; ^2^ Beijing Institute for Stem Cell and Regenerative Medicine Beijing China; ^3^ Senior Department of Respiratory and Critical Care Medicine The Eighth Medical Center of Chinese PLA General Hospital Beijing China; ^4^ National Center for Respiratory Medicine; State Key Laboratory of Respiratory Health and Multimorbidity; National Clinical Research Center for Respiratory Diseases Beijing China; ^5^ Institute of Respiratory Medicine Chinese Academy of Medical Sciences and Peking Union Medical College Beijing China; ^6^ Department of Pulmonary and Critical Care Medicine, Center of Respiratory Medicine China‐Japan Friendship Hospital Beijing China; ^7^ University of Chinese Academy of Sciences Beijing China; ^8^ Chinese Academy of Medical Sciences & Peking Union Medical College Beijing China; ^9^ Institute of Clinical Medical Sciences China‐Japan Friendship Hospital Beijing China; ^10^ Tianjin Key Laboratory of Lung Regenerative Medicine, Haihe Hospital Tianjin University Tianjin China; ^11^ Key Research Laboratory for Infectious Disease Prevention for State Administration of Traditional Chinese Medicine Tianjin Institute of Respiratory Diseases Tianjin China; ^12^ Respiratory Research Institute, Senior Department of Pulmonary & Critical Care Medicine The 8th Medical Center of PLA General Hospital Beijing China; ^13^ Beijing Key Laboratory for Immune‐Mediated Inflammatory Diseases, Institute of Medical Science China‐Japan Friendship Hospital Beijing China; ^14^ National Stem Cell Resource Center, Institute of Zoology Chinese Academy of Sciences Beijing China

**Keywords:** excessive extracellular matrix, idiopathic pulmonary fibrosis, IMRCs, MMP1

## Abstract

Currently, no targeted therapy exists for idiopathic pulmonary fibrosis (IPF). The hallmark pathological feature of excessive extracellular matrix (ECM) deposition severely undermines the efficacy of mesenchymal stem cell (MSC)‐based treatments. While existing MSC therapeutic strategies primarily focus on modulating inflammation in early stages, they have not yet established precise interventions addressing the core pathological mechanism—ECM dysregulation. Previous studies demonstrated the therapeutic potential of human embryonic stem cell (hESCs)‐derived immunity‐and‐matrix‐regulatory cells (IMRCs) in lung injury and fibrosis models. However, the critical biomarkers and underlying mechanisms mediating IMRCs' efficacy in IPF remain poorly understood. In this study, we generated *MMP1* knockout IMRCs (IMRCs‐*MMP1 KO*) using CRISPR‐based gene editing. We then characterized whether MMP1 ablation affected key properties of IMRCs, including cell morphology, proliferation, migration, marker protein expression, transcriptomic profile, and cytokine secretion. Subsequently, the ability of IMRCs‐*MMP1 KO* to degrade collagen was tested using in vivo and in vitro pulmonary fibrosis models. *MMP1* knockout was successfully achieved and did not compromise typical IMRC characteristics or impair their immunomodulatory capacity. However, MMP1 deficiency significantly attenuated the ability of IMRCs to degrade TGF‐β1‐induced collagen I deposition in A549 cells. Importantly, wild‐type IMRCs demonstrated superior therapeutic efficacy in ameliorating bleomycin‐induced lung injury and fibrosis in mice compared with IMRCs‐*MMP1 KO*. Furthermore, IMRCs exhibited significantly greater capability to directly degrade the pericellular collagen I and modulate fibroblasts' activation progression within fibrotic lung tissues in a MMP1‐dependent manner. In summary, our data establish that MMP1 plays an essential functional role in IMRC‐mediated attenuation of PF. MMP1 thus represents a key therapeutic biomarker for IMRC‐based treatment. This work provides a foundation for developing stem cell therapies tailored to the pathological features of IPF, potentially enabling adaptive treatment strategies.

## Graphical abstract

Human embryonic stem cell‐derived Immunity‐and‐Matrix‐Regulatory Cells (IMRCs) effectively attenuate pulmonary fibrosis by secreting Matrix Metalloproteinase‐1 (MMP1), which directly degrades excess collagen I in the extracellular matrix. By directly degrading collagen I, IMRCs reverse ECM stiffness, suppress myofibroblast activation, and promote a transitional fibroblast state, offering a novel strategy against IPF beyond immunomodulation.

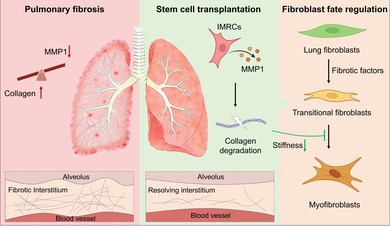

## 1 Introduction

Pulmonary fibrosis (PF), a fatal fibrotic disease that is difficult to cure, represents a major end‐stage pathological change in chronic lung disorders, particularly idiopathic pulmonary fibrosis (IPF) [1]. Prognosis is gravely dismal, with median survival of only 2–5 years post‐diagnosis [2, 3]. IPF is a chronic, progressive respiratory condition characterized by pulmonary interstitial fibrosis leading to lung tissue thickening and sclerosis. This pathology culminates in progressive, irreversible lung function decline [4]. Globally, ~5 million individuals suffer from IPF, with incidence rates ranging from 10 to 60 per 100,000 people—alarmingly increasing by ~11% annually [3, 5, 6]. Currently, no definitive pharmacological treatments exist for IPF. Lung transplantation remains the most effective option, yet its clinical application is severely limited by donor organ scarcity, procedural costs, and a modest post‐transplant five‐year survival increase of only ~45% [7]. Currently, only two drugs, pirfenidone and nintedanib, have been approved for clinical use in IPF management. However, these drugs fail to improve survival rates and are associated with significant side effects [8, 9]. Consequently, developing targeted IPF therapeutics is critically urgent.

Stem cells are renowned for their capacity for self‐renewal and multi‐directional differentiation, endowing them with significant clinical potential, particularly for treating life‐threatening fibrotic diseases like IPF. Numerous preclinical studies have demonstrated that intravenous administration of mesenchymal stem cells (MSCs), either derived from tissue or human pluripotent stem cells (hPSCs), can migrate to sites of lung injury, suppressing inflammation, promoting tissue repair, and mitigating PF [10, 11, 12, 13]. While MSCs exhibit favourable clinical safety profiles, substantial heterogeneity in their clinical efficacy persists across studies [14, 15, 16, 17, 18, 19, 20, 21]. Most clinical trials in IPF patients indicate that MSC therapy primarily stabilizes or delays the progression of fibrosis. However, its impact on improving lung function and quality of life for patients with end‐stage IPF remains limited. Critically, MSC therapy has not yet demonstrated significant efficacy in inhibiting the relentless disease progression.

Compared with promising preclinical results, the clinical trial outcomes for MSCs in PF have been disappointing. This disparity likely stems from critical differences in the pathological status of the recipient microenvironment. Most preclinical studies predominantly administer MSCs during the early inflammatory phase of the disease, rather than the later fibrotic stage. During this phase, MSCs effectively suppress inflammation and thereby attenuate PF progression [22, 23]. However, their therapeutic efficacy diminishes significantly when applied to advanced fibrotic disease. The importance of early post‐injury MSC transplantation has been corroborated by other studies [24, 25, 26]. IPF itself is pathologically characterized by excessive extracellular matrix (ECM) deposition and the formation of irreversible fibrotic scars within the distal lung parenchyma [7]. This progressive scarring leads to a gradual decline in lung function and quality of life, culminating in respiratory failure and death. Consequently, the limited clinical efficacy observed when MSCs are administered during the established fibrosis phase may be attributed to the hostile, densely fibrotic microenvironment. Therefore, novel strategies are urgently needed to enhance the therapeutic potential of MSCs. These strategies must enable MSCs not only to halt the initiation and progression of IPF but also to actively remodel fibrotic lesions and restore functional lung tissue.

In previous study, human embryonic stem cells (hESCs)‐derived immunity‐and‐matrix‐regulatory cells (IMRCs) have been generated, which have demonstrated therapeutic efficacy in murine model of bleomycin‐induced lung injury [27]. Furthermore, IMRCs have been clinically evaluated for treating critical COVID‐19 illness (NCT04331613) [28] and PF (ChiCTR2000031139) [29] in patients following severe acute respiratory syndrome coronavirus 2 (SARS‐CoV‐2) infection. Extracellular vesicles derived from IMRCs have also shown effectiveness in treating murine PF [30]. Nevertheless, the key biomarkers and precise molecular mechanisms underlying IMRCs' therapeutic effects against IPF remain incompletely characterized.

Interestingly, IMRCs exhibit significantly higher matrix metalloproteinase 1 (MMP1) expression than primary umbilical cord mesenchymal stem cells (UCMSCs) [27]. As a zinc‐dependent protease within the MMP family, MMP1 is predominantly secreted by stromal cells [31, 32]. Its primary function involves degrading types I, II, and III collagen, contributing to multiple physiological processes including extracellular matrix remodelling. Significantly, transplantation of bone marrow‐derived MSCs (BM‐MSCs) genetically modified to overexpress MMP1 reduced collagen deposition in fibrotic liver tissue, inhibited hepatic stellate cell activation, and consequently attenuated liver injury and fibrosis [33]. Crucially, conditioned medium from IMRCs was shown to suppress TGF‐β1‐induced collagen I expression [27]. Therefore, MMP1 may play an important role in IMRC efficacy for IPF treatment.

In this study, we generated *MMP1* knockout IMRCs (IMRCs‐*MMP1 KO*) using CRISPR‐Cas9 technology. While MMP1 ablation did not alter fundamental IMRC characteristics, it significantly impaired their ability to degrade collagen I in vitro. Crucially, intravenous administration of IMRCs‐*MMP1 KO* resulted in substantially worse therapeutic outcomes relative to wild‐type IMRCs. This included diminished preservation of alveolar type II epithelial cells and markedly reduced collagen I degradation in treated animals. This study will provide a basis for developing an adaptive treatment strategy for stem cells tailored to the pathological characteristics of IPF.

## 2 Materials and Methods

### 2.1 Ethics Statement

All animal experiments were approved by the Animal Research Laboratory Animal Welfare Ethics Review Committee of Institute of Zoology, Chinese Academy of Sciences (Title: Study on the Therapeutic Mechanisms of Mesenchymal‐like Stem Cells for Pulmonary Fibrosis; Date: 18.04.2022; Number: IOZ‐IACUC‐2021‐031). The hESC line was provided by the National Stem Cell Resource Center (NSCRC; http://www.nscrc.cn/) and published in Stem Cell Reports (DOI: https://doi.org/10.1016/j.stemcr.2017.04.017). The NSCRC confirmed that there was initial ethical approval for collection of human cells, and the donors had signed informed consent. No written consent has been obtained from the patients as there is no patient‐identifiable data included.

### 2.2 IMRCs Harvesting and Culture

The IMRCs were prepared according to previously established protocols [27]. Briefly, the IMRCs were derived from human embryoid bodies (hEBs) by passaging migratory cells with serum‐free reagents. The clinical hESC line, Q‐CTS‐hESC‐2, provided by the National Stem Cell Resource Center (NSCRC), Institute of Zoology, Chinese Academy of Sciences (CAS), was prepared as described previously [34]. To generate hEBs, hESCs were dissociated into small clumps and cultured for 5 days. Subsequently, the hEBs were transferred onto vitronectin‐coated plates and cultured for an additional 14 days. During this period, the outgrowth occurred from the hEBs. These outgrowth cells were dissociated and passaged in “IMRCs Medium”. After 5 passages in culture, IMRCs were harvested, exhibiting a fibroblastic morphology. To prepare the conditioned medium of IMRCs, the cells were cultured to 50% confluence in culture dishes and then transferred to fresh medium. After 24 h of incubation, the conditioned medium was collected. All cultures were maintained at 37°C, 5% CO_2_ and atmospheric O_2_ in a humidified incubator (Thermo Fisher Scientific, Waltham, MA, USA).

### 2.3 The Generation of *MMP1* Knockout IMRCs Cell Lines

Initially, a sgRNA was designed specifically to target the first exon of the human *MMP1* gene. Then, the *MMP1* sgRNA was constructed into the CRISPR‐Cas9 plasmid purchased from HANBIO (Beijing, China). Utilizing the P3 Primary Cell Nucleofection Kit (Lonza, Basel, Switzerland; V4XP‐3024), the CRISPR‐Cas9‐*MMP1*‐sgRNA plasmid was electroporated into hESCs. Post‐electroporation, single cell clones were cultured and collected to establish *MMP1* knockout hESCs lines for analysis and functional studies. Subsequently, the *MMP1* knockout hESCs line was used to generate IMRCs (IMRCs‐*MMP1 KO*) by the established differentiation system. *MMP1* sgRNA: AGCAAATGCAGGAATTCTTTGGG.

### 2.4 Animals

Eleven to thirteen‐week‐old C57BL/6J male specific pathogen‐free (SPF) mice were purchased from the Vital River (Beijing, China). To ensure consistency, all experimental and control mice were carefully selected to have weights ranging from 25 to 30 g. All mice were housed in the animal care facility in the Institute of Zoology, Chinese Academy of Sciences. Mice were maintained under SPF conditions at room temperature (between 20°C and 24°C), with humidity between 35% and 55%, in a 12/12 h light/dark cycle, with food and water ad libitum. Their general health, fur condition, activity levels, and weights were monitored regularly in accordance with institutional guidelines. At each experimental endpoint, mice were sacrificed by CO_2_ euthanasia. No blinding was performed in this animal study. The Animal Research Laboratory Animal Welfare Ethics Review Committee of the Institute of Zoology, Chinese Academy of Sciences approved all procedures (IOZ‐IACUC‐2021‐031). The work has been reported in line with the ARRIVE guidelines 2.0.

### 2.5 Lung Fibrosis and Cells Transplantation

The lung fibrosis mouse model was generated as described previously [27]. Briefly, mice were injected intratracheally with 1.5 mg·kg^−1^ bleomycin sulphate (BLM; Bioway, China; DP721) in normal saline under light anaesthesia. The 1.65 × 10^8^/kg·wight IMRCs or IMRCs‐*MMP1 KO* were delivered intravenously at day 1 or day 7 after injury. The lung function was detected and mice were euthanized at day 21 after BLM injury. After perfusion with normal saline, the left lungs were used for morphometric analysis, and the right lungs were removed and used for other analyses.

### 2.6 Single‐Cell RNA Sequencing Libraries Preparation and Sequencing

To prepare single‐cell suspensions of mouse lung tissue, mice (*n* = 3, per group) were euthanized on day 9 after bleomycin injury and subjected to pulmonary perfusion with PBS. All samples from each group were digested using the Mouse Lung Dissociation Kit (Miltenyi Biotec, Cologne, Germany; 130‐095‐927) according to the manufacturer's instructions. Following digestion, cells were sequentially filtered through 70 μm and 40 μm cell strainers to remove tissue debris, followed by erythrocyte depletion using Red Blood Cell Lysis Buffer (Miltenyi Biotech, 130‐094‐183). Single‐cell RNA sequencing libraries were constructed using the Chromium Next GEM Single Cell 5′ GEM, Library & Gel Bead Kit V2 (10× Genomics, California, USA). Libraries underwent 150 bp paired‐end sequencing on the NovaSeq X Plus platform (Illumina). Raw sequencing data were demultiplexed, aligned to the mouse reference genome (mm 10), and quantified using Cell Ranger (10 × Genomics) to generate gene expression matrices. Subsequent bioinformatic analyses, including UMAP visualization, cluster identification, and gene expression mapping, were performed using the R package Seurat v5. Cell type specific marker genes are detailed in Supporting Information, (Table S2).

### 2.7 Statistics

All data are expressed as mean ± SEM. Survival curves were derived by the Kaplan–Meier method and compared via generalized Wilcoxon test. Statistical analysis was performed using GraphPad Prism 9.0 statistical software (San Diego, CA, USA). The statistical significance of multiple groups was compared with each other using Tukey's multiple comparison test ANOVA. A *p* value of < 0.05 was considered statistically significant.

## 3 Results

### 3.1 Generation of *MMP1* Knockout IMRCs

**FIGURE 1:**
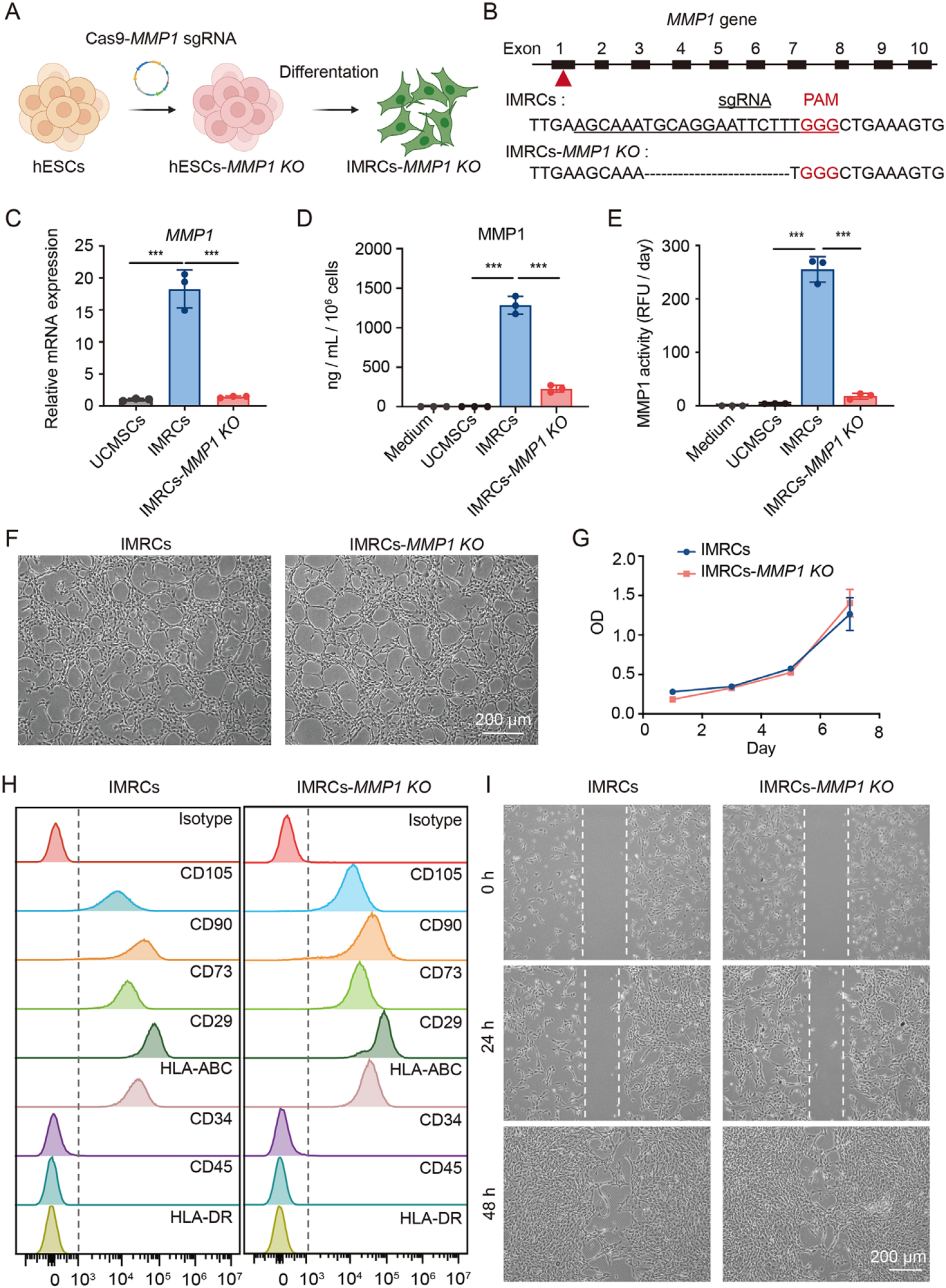
Generation and characterization of *MMP1* knockout Immunity‐and Matrix‐Regulatory Cells (IMRCs‐*MMP1 KO*). (A) Schematic representation of the various phases involved in the derivation protocol for IMRCs‐*MMP1 KO*. (B) Representative morphology of cells at different stages observed by phase contrast microscopy. hEBs, human embryoid bodies. (C) Quantitative PCR analysis revealing the expression levels of *MMP1* mRNA in UCMSCs, IMRCs and IMRCs‐*MMP1 KO*. UCMSCs, umbilical cord mesenchymal stem cells. (D) ELISA for MMP1 protein in the conditioned media of UCMSCs, IMRCs, IMRCs‐*MMP1 KO* and fresh medium. (E) Assessment of MMP1 activity in the conditioned media of UCMSCs, IMRCs, IMRCs‐*MMP1 KO* and fresh medium. (F) Representative morphology of IMRCs and IMRCs‐*MMP1 KO* observed by phase contrast microscopy. (G) Proliferation curve of IMRCs and IMRCs‐*MMP1 KO* over time. (H) The expression of MSC‐specific surface markers in IMRCs‐*MMP1 KO* was determined by flow cytometry. Isotype control antibodies were used as controls for gating. (I) The width of the scratch was observed under an inverted optical microscope at 0, 24 and 48 h post‐scratch, comparing the healing progress of IMRCs and IMRCs‐*MMP1 KO* cultures. *p < 0.05, **p < 0.01, ***p < 0.001; data are represented as the mean ± SEM, *n* = 3.

To elucidate the functional role of *MMP1* in IMRCs, IMRCs‐*MMP1* knockout cells (IMRCs‐*MMP1 KO*) were generated by the CRISPR‐Cas9 system (Figure 1A). First, a CRISPR‐Cas9 plasmid targeting *MMP1* with sgRNA was transduced into hESCs to establish *MMP1*‐KO hESC lines. Positive clones were then differentiated into IMRCs*‐MMP1 KO* using established protocols [27]. Sequencing analysis revealed a 13‐base deletion in exon 1 of IMRCs‐*MMP1 KO* compared with wild‐type IMRCs (Figure 1B; Supporting Information, Figure S1A). Consistent with this, RT‐qPCR showed significantly reduced *MMP1* expression in IMRCs‐*MMP1 KO* relative to wild‐type IMRCs (Figure 1C; Supporting Information, Table S1). Similarly, ELISA of conditioned media and western blot demonstrated lower MMP1 protein levels (Figure 1D; Supporting Information, Figure S1B,C) and reduced enzymatic activity (Figure 1E) in IMRCs‐*MMP1 KO*. These results indicated *MMP1* gene was successfully knockout in IMRCs‐*MMP1 KO*. Next, the characteristics of IMRCs‐*MMP1 KO* were analysed. Both IMRCs and IMRCs‐*MMP1 KO* displayed similar cell morphology and proliferation rates (Figure 1F,G). Flow cytometry analysis further confirmed IMRCs‐*MMP1 KO* retained the characteristic surface marker profile as IMRCs: positive for CD105, CD90, CD73, CD29 and HLA‐ABC, and negative for CD45, CD34 and HLA‐DR (Figure 1H). Given reports implicating MMP1 in cell migration [35, 36, 37], we assessed the migratory capacity of IMRCs‐*MMP1 KO* using a scratch assay. This result revealed that IMRCs‐*MMP1 KO* displayed similar migration ability to IMRCs (Figure 1I; Supporting Information, Figure S1D). Collectively, these data demonstrate successful *MMP1* knockout without compromising key IMRC characteristics.

### 3.2 IMRCs‐ *MMP1 KO* Possess Similar Gene Expression Characteristics to IMRCs

**FIGURE 2:**
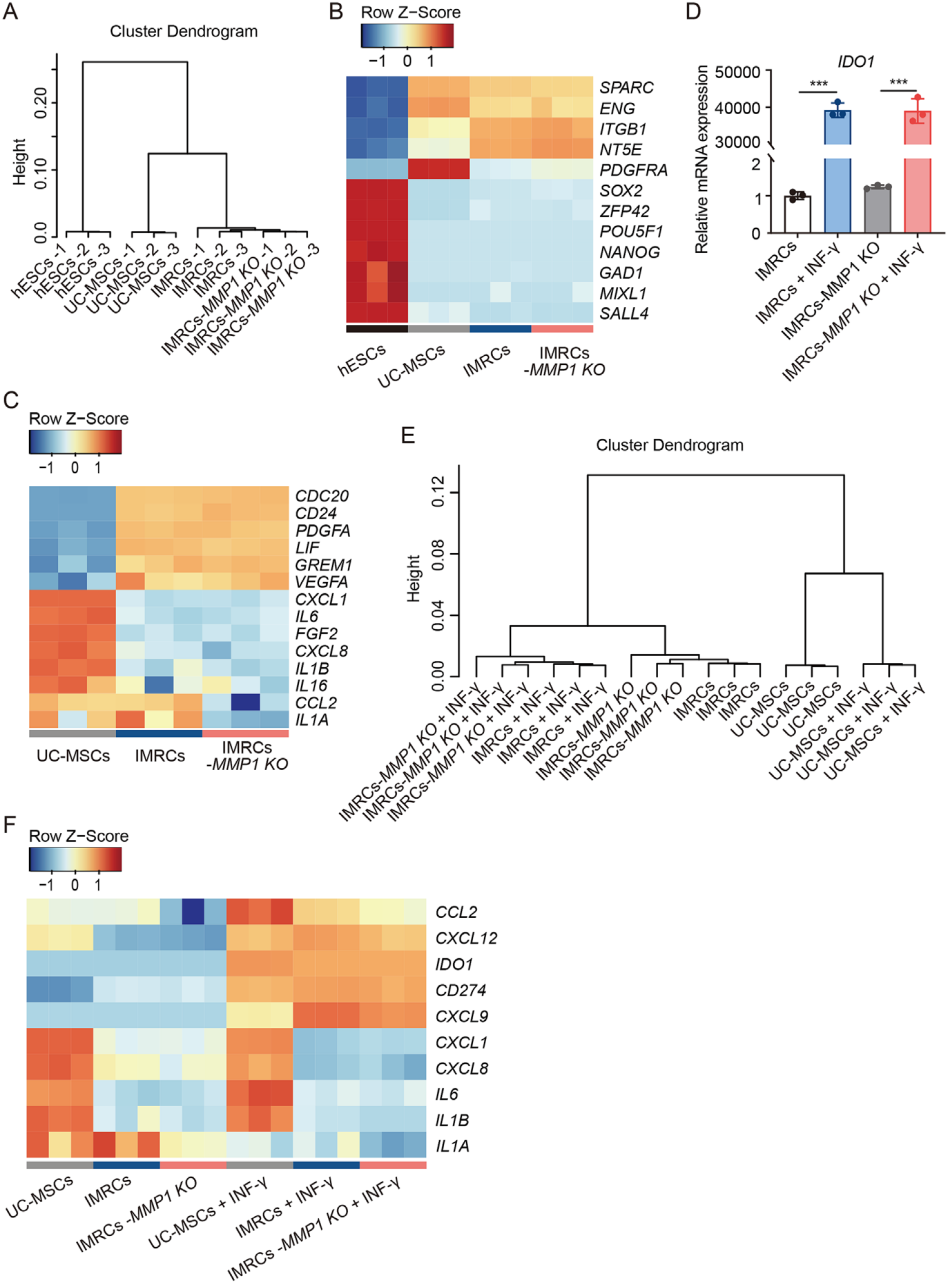
*MMP1* knockout doesn't affect IMRCs' gene expression characteristics. (A) Unsupervised hierarchical clustering analysis of IMRCs, IMRCs‐*MMP1 KO*, UCMSCs and hESCs. (B) Heatmap showing MSC‐specific marker and pluripotency marker gene expression. (C) Quantitative PCR for *IDO1* mRNA in IMRCs and IMRCs‐*MMP1 KO* before and after 100 ng/mL IFN‐γ treatment. *GAPDH* was used for internal normalization. (D) Heatmap displaying the differentially expressed genes in IMRCs, IMRCs‐*MMP1 KO* and UCMSCs; *p < 0.05, **p < 0.01, ***p < 0.001; data are represented as the mean ± SEM, *n* = 3. (E) Unsupervised hierarchical clustering analysis of IMRCs, IMRCs‐*MMP1 KO* and UCMSCs before and after IFN‐γ stimulation. (F) Heatmap illustrating the expression of cytokines in IMRCs, IMRCs‐*MMP1 KO* and UCMSCs before and after IFN‐γ stimulation.

To evaluate global transcriptional similarity between IMRCs and IMRCs‐*MMP1 KO*, we conducted whole‐transcriptome profiling and compared their gene expression patterns with hESCs and UCMSCs (Supporting Information, Table S3). Unsupervised hierarchical clustering revealed that IMRCs‐*MMP1 KO* clustered closely with IMRCs, indicating high transcriptomic similarity (Figure 2A). Analysis of MSC‐specific genes further showed that IMRCs‐*MMP1 KO* exhibited expression patterns analogous to both IMRCs and UCMSCs but distinct from hESCs (Figure 2B). Notably, both IMRCs and IMRCs‐*MMP1 KO* lacked expression of all pluripotency genes (*POU5F1*, *NANOG*, *SOX2*), while expressing MSC‐specific markers (*SPARC*, *NT5E*, *ENG*, and *ITGB1*). Additionally, we also observed differential expression of numerous genes between IMRCs and IMRCs‐*MMP1 KO* compared with primary UCMSCs. Upregulated genes included immunomodulatory (*LIF* and *CD24*), tissue repair (*VEGFA*, *GREM1*, and *PDGFA*), and cell division (*CDC20*) (Figure 2C). Conversely, pro‐inflammatory genes (*IL6*, *IL16*, *IL1B*, *CXCL8*, *CCL2*, and *CXCL1*) were consistently downregulated (Figure 2C). To further investigate the immunomodulatory capacity, IMRCs and IMRCs‐*MMP1 KO* were exposed to the 100 ng/mL pro‐inflammatory cytokine IFN‐γ for 24 h [38, 39, 40]. After stimulation with IFN‐γ, both IMRCs and IMRCs‐*MMP1 KO* displayed similar upregulated expression of *IDO1* (Figure 2D). Hierarchical clustering confirmed that IFN‐γ‐stimulated IMRCs‐*MMP1 KO* clustered separately from UCMSCs and aligned closely with stimulated IMRCs (Figure 2E). Global gene expression analysis further demonstrated that compared with primary UCMSCs, both IMRCs and IMRCs‐*MMP1 KO* showed lower expression of pro‐inflammatory genes and higher expression of anti‐inflammatory genes (Figure 2F). This finding was consistent with the cytokine analysis, which revealed that IMRCs‐*MMP1 KO* displayed similar exocrine abilities to IMRCs regardless of IFN‐γ treatment (Supporting Information, Figure S2). These results indicated that MMP1 deletion does not compromise the immunoregulatory functions characteristic of IMRCs.

### 3.3 MMP1 Knockout Profoundly Impairs Collagen I Degradation by IMRCs In Vitro

**FIGURE 3:**
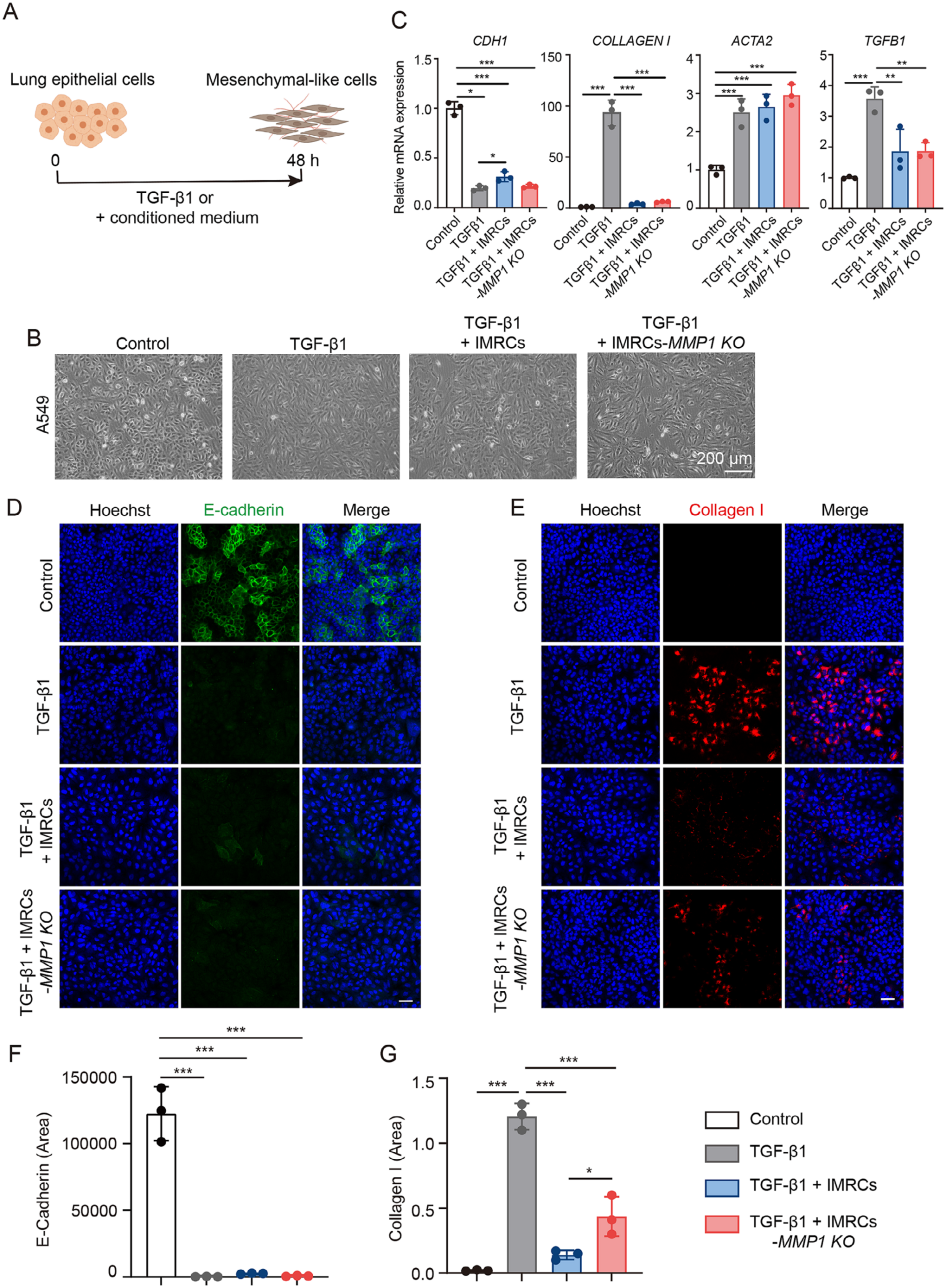
Knockout of *MMP1* affects the capacity of IMRCs to degrade collagen I induced by TGF‐β1 in vitro. (A) Diagram of the in vitro epithelial‐mesenchymal transition (EMT) protocol induced by TGF‐β1 with or without conditioned medium for 48 h in A549 cells. (B) Representative morphology of A549 cells treated with: No TGF‐β1 (Control), 10 ng/mL TGF‐β1 (TGF‐β1), 10 ng/mL TGF‐β1 plus conditioned medium from IMRCs (TGF‐β1 + IMRCs), and 10 ng/mL TGF‐β1 plus conditioned medium from IMRCs‐*MMP1 KO* (TGF‐β1 + IMRCs‐*MMP1 KO*) for 48 h. *p < 0.05, **p < 0.01, ***p < 0.001; data are represented as the mean ± SEM, *n* = 3. (C) Quantitative PCR for the relative expression of *CDH1*, *COLLAGEN I*, *ACTA2* and *TGFB1* mRNA in A549 cells with different treatment for 48 h. (D) Immunofluorescence staining for E‐cadherin expression in A549 cells with different conditions for 48 h. Scale bar, 50 μm. (E) Immunofluorescence staining for Collagen I expression in A549 cells following 48 h of treatment. Scale bar, 50 μm. (F) Quantification of the relative E‐cadherin protein expression levels in (D). (G) Quantification of the relative Collagen I protein expression levels in (E). *p < 0.05, ***p < 0.001; data are represented as the mean ± SEM, *n* = 3.

To investigate the role of the *MMP1* in the anti‐fibrotic function of IMRCs, the human epithelial cell line A549 was used to establish an in vitro epithelial‐mesenchymal transition (EMT) model (Figure 3A). Following 48‐h treatment with 10 ng/mL TGF‐β1, A549 cells exhibited a mesenchymal‐like morphology (Figure 3B). Conditioned media from either wild‐type IMRCs or IMRCs‐*MMP1 KO*, added concurrently with TGF‐β1, did not prevent this morphological transformation (Figure 3B). The analysis of qPCR showed that both IMRCs and IMRCs‐*MMP1 KO* conditioned media significantly reduced TGF‐β1‐induced upregulation of *COL1A1* and *TGFB1* expression (Figure 3C; Supporting Information, Table S1). Consistent with previous results, *CDH1* expression remained unchanged regardless of conditioned media treatment (Figure 3C,D,F). We next assessed collagen clearance capacity. Thereafter, A549 cells were treated with TGF‐β1 for 48 h and co‐cultured with the conditioned media of IMRCs or IMRCs‐*MMP1 KO*. Immunofluorescence analyses demonstrated markedly stronger reduction of collagen I protein expression by IMRCs conditioned media compared with IMRCs‐*MMP1 KO* conditioned media (Figure 3E,G). Although the A549 EMT model can simulate some extracellular matrix secretion, it has limitations in modelling pulmonary fibrosis, in which myofibroblasts are the primary source of the extracellular matrix. To specifically assess the impact of IMRCs on fibroblast activation, we employed human lung fibroblasts (MRC‐5 cells) stimulated with TGF‐β1. MRC‐5 cells were treated with TGF‐β1 to induce a profibrotic phenotype (Figure S3A). These activated fibroblasts were then co‐cultured with wild‐type IMRCs or IMRCs‐*MMP1 KO*. Western blot analysis showed that TGF‐β1 treatment markedly upregulated the protein expression of collagen I and α‐SMA in MRC‐5 cells (Figure S3B,C). Co‐culture with wild‐type IMRCs significantly reduced both collagen I and α‐SMA levels (Figure S3B,C). Critically, this anti‐fibrotic effect was abolished in the IMRCs‐*MMP1 KO* group, where collagen I and α‐SMA levels remained high, similar to the TGF‐β1‐only control (Figure S3B,C). These results suggested that MMP1 knockout critically impairs the ability of IMRCs to degrade deposited.

### 3.4 IMRCs Ameliorate Lung Fibrosis After Injury in a MMP1‐Dependent Manner

**FIGURE 4:**
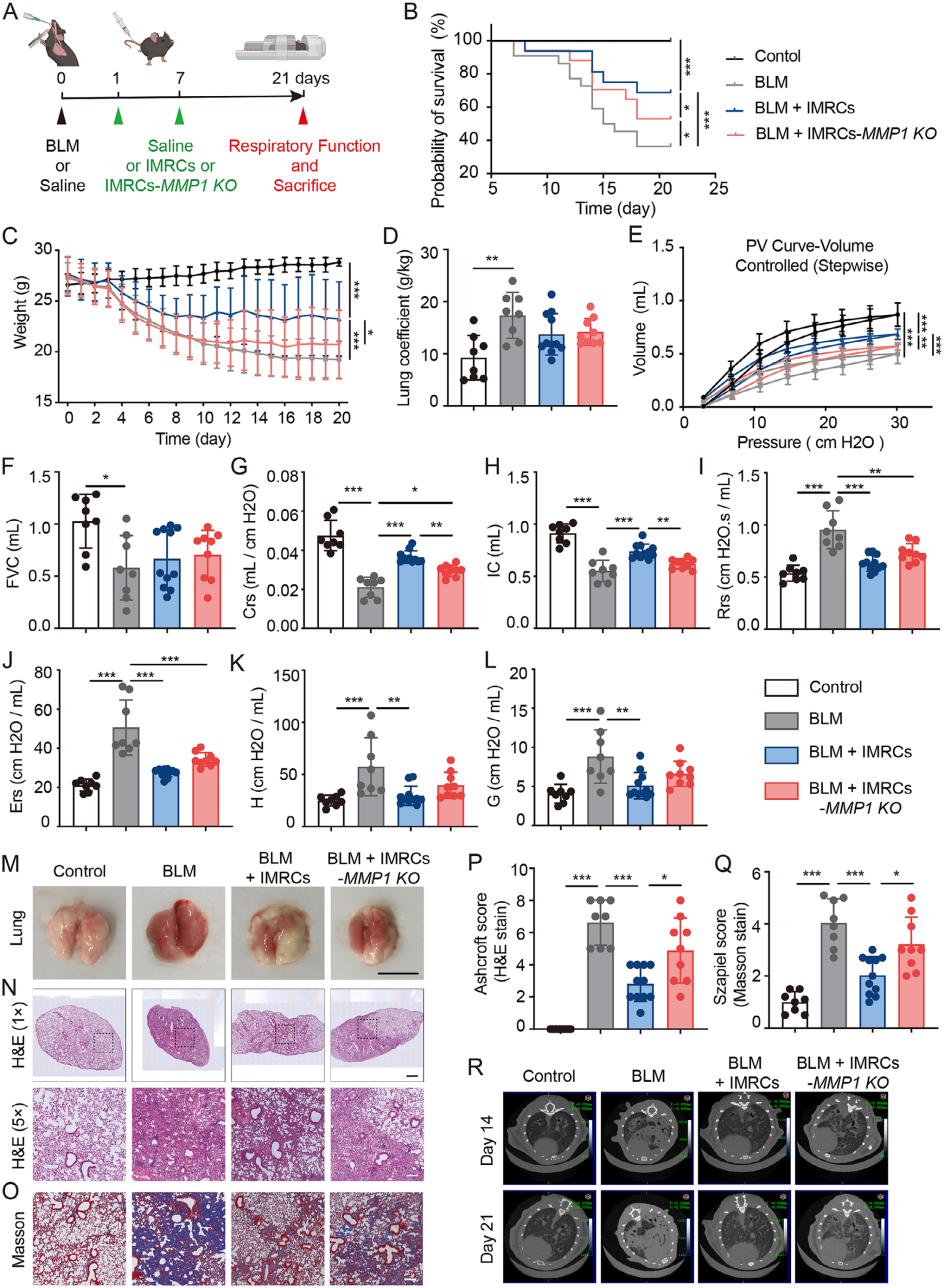
Knockout of *MMP1* reduces the efficacy of IMRCs in treating lung fibrosis. (A) Diagram of the animal experimental protocol for lung fibrosis. Mice were randomly assigned to the four experimental groups with consistent baseline body weights using a computer‐generated random number table. Mice were administered intratracheal bleomycin (BLM; 2.5 mg/kg · body weight) or the equivalent volume of saline on day 0. On day 1 and 7, some BLM‐injured mice were administered an intravenous injection of IMRCs or IMRCs‐*MMP1 KO* (1.65 × 10^8^ cells/kg · body wight) (BLM + IMRCs, *n* = 16 and BLM + IMRCs‐*MMP1 KO* group, *n* = 17) via the caudal vein. BLM‐injured (BLM, *n* = 22) and normal control (Control, *n* = 8) mice received the equivalent volume of saline. Mice were randomly assigned to these groups. (B) Kaplan–Meier survival curves illustrated the survival rates of mice subjected to various interventions. (C) The body weight changes of the mice receiving different interventions. (D) The lung coefficient was calculated as the ratio of wet lung weight to total body weight. (E–L) Lung mechanical function was measured by FlexiVent on day 21, including PV curves, FVC, Crs, IC, Rrs, Ers, (H) and (G) for all the experimental groups. PV, pressure‐volume; FVC, forced vital capacity; Crs, static compliance; IC, inspiratory capacity; Rrs, respiratory resistance; Ers, elastic resistance; H, tissue elasticity; G, tissue damping. M Representative images of whole lungs from all groups on day 21 post‐injury. Scale bar, 1 cm. N Representative histology of lung sections stained with haematoxylin and eosin (H&E) (Scale bar, 1 mm and 200 μm) on day 21 post‐injury. O Representative lung sections of all groups stained with Masson's trichrome on day 21 post‐injury. Scale bar, 200 μm. P Quantitative analysis of fibrotic changes with the Ashcroft score based on the lung H&E sections. The mean score of severity in six microscopic fields per section was calculated. Q Fibrotic changes are also evaluated using the Szappiel scoring system on lung sections stained with Masson's trichrome. R Micro‐computed tomography (micro‐CT) scans of the mouse lungs from all the experimental groups were presented on day 14 and 21. (C–Q) Control, *n* = 8; BLM, *n* = 8; BLM + IMRCs, *n* = 11 and BLM + IMRCs‐*MMP1 KO*, *n* = 9. *p < 0.05, **p < 0.01, ***p < 0.001; data are represented as the mean ± SEM.

To assess the therapeutic efficacy of IMRCs‐*MMP1 KO* against lung fibrosis, cells were administered intravenously into mice with BLM‐induced lung injury based on our previous study [41] (Figure 4A). Kaplan–Meier survival curves revealed that IMRCs treatment significantly improved survival rates in BLM‐exposed mice (BLM group 40.0% vs. IMRCs 68.8%, IMRCs‐*MMP1 KO* 52.9%), indicating MMP1‐dependent protection (Figure 4B). Additionally, IMRCs also attenuated BLM‐induced body weight loss more effectively than IMRCs‐*MMP1 KO* (Figure 4C). Pulmonary edema showed only marginal improvement with either treatment (Figure 4D). Functional assessments revealed that IMRCs conferred the most substantial recovery of lung capacity metrics, including pressure‐volume (PV), forced vital capacity (FVC), static compliance (Crs), and inspiratory capacity (IC) (Figure 4E–H). Furthermore, IMRCs treatment also resulted in the greatest reduction in functional indices for lung fibrosis, including respiratory resistance (Rrs), elastic resistance (Ers), tissue elasticity (H), and tissue damping (G) (Figure 4I–L). Histopathological analysis confirmed that IMRCs treatment significantly improved lung architecture and reduced alveolar thickening in a MMP1‐dependent manner (Figure 4M–O). Consistent with this, Ashcroft and Szapiel fibrosis scores showed MMP1‐dependent attenuation following IMRCs treatment (Figure 4P,Q). Computed tomography (CT) imaging also showed superior structural recovery with IMRCs compared with IMRCs‐*MMP1 KO* (Figure 4R). Collectively, these results suggested that IMRCs mitigate post‐injury lung fibrosis primarily through MMP1‐mediated mechanisms.

### 3.5 IMRCs Directly Degrade Collagen I Through MMP1 Secretion In Vivo

**FIGURE 5:**
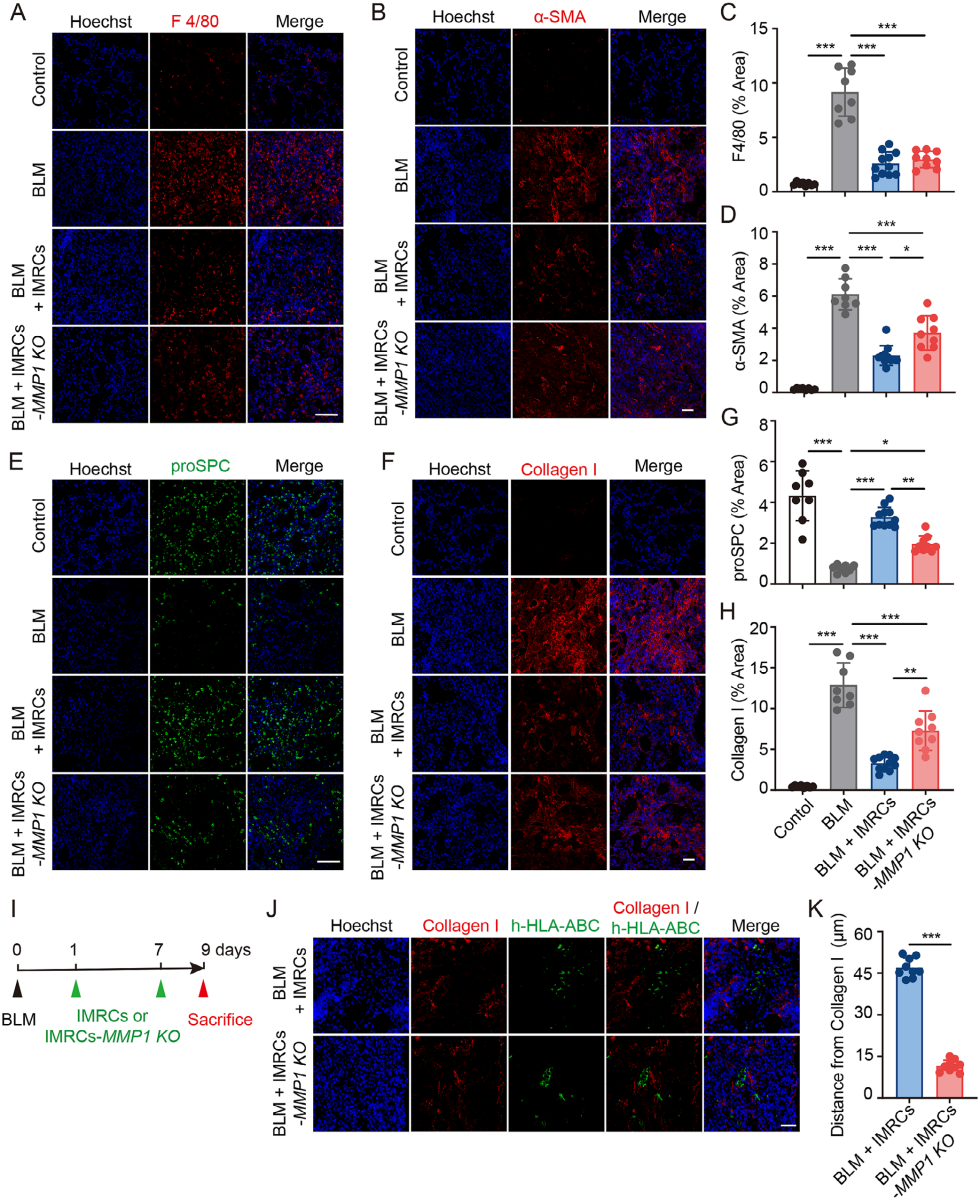
The direct degradation of surrounding Collagen I by IMRCs in lung fibrosis mice via secreting MMP1. (A) Immunofluorescent analysis of F4/80 protein expression in mice subjected to various interventions. Scale bars, 100 μm. (B) Immunofluorescent staining for the protein expression of α‐SMA in mice receiving different interventions. Scale bars, 50 μm. (C) Quantification of the F4/80 protein expression levels shown in A. (D) Quantification of the α‐SMA protein expression levels shown in (B). (E) Immunofluorescent staining to assess proSPC protein expression in mice under different interventions. Scale bars, 100 μm. (F) Immunofluorescent staining of Collagen I protein expression in mice receiving various interventions. Scale bars, 50 μm. (G) Evaluation of alveolar type II cells (AT II) by proSPC area in (E). (H) Quantification of the Collagen I protein expression levels in (F). (I) Diagram of the animal experimental protocol. On day 9, some mice received IMRCs or IMRCs‐*MMP1 KO* injection to assess the effect of *MMP1* knockout in vivo. (J) Immunofluorescent staining for Collagen I (red) in mice injected with IMRCs or IMRCs‐*MMP1 KO*. IMRCs and IMRCs‐*MMP1 KO* were labelled with anti‐human HLA‐ABC antibody (green). Scale bars, 50 μm. (K), Quantification of the distance between Collagen I and IMRCs or IMRCs‐*MMP1 KO*. (C–H) Control, *n* = 8; BLM, *n* = 8; BLM + IMRCs, *n* = 11 and BLM + IMRCs‐*MMP1 KO*, *n* = 9. *p < 0.05, **p < 0.01, ***p < 0.001; data are represented as the mean ± SEM.

Immunofluorescence analyses showed that administration of either IMRCs or IMRCs‐*MMP1 KO* significantly reduced F4/80^+^ macrophage infiltration at lung injury sites compared with BLM‐only controls, indicating MMP1‐independent anti‐inflammatory effects (Figure 5A,C). Strikingly, α‐SMA^+^ myofibroblast accumulation was markedly attenuated in IMRCs‐treated mice versus IMRCs‐*MMP1 KO* recipients, suggesting MMP1‐dependent inhibition of myofibroblast activity (Figure 5B,D). Notably, wild‐type IMRCs also significantly enhanced alveolar type II cell regeneration, evidenced by elevated pro‐SPC expression relative to IMRCs‐*MMP1 KO*, indicating that *MMP1* knockout diminished the regenerative capacity of alveolar type II cells enhanced by IMRCs infusion (Figure 5E,G). Correspondingly, collagen I deposition was substantially lower in wild‐type IMRC‐treated lungs than in IMRCs‐*MMP1 KO* counterparts (Figure 5F,H; Supporting Information, Figure S4). Based on previous IMRCs survival data in vivo [27], IMRCs were rapidly cleared within 5 days after injection. To investigate the direct interaction between IMRCs and collagen I during the peak window of cells activity, the lung tissues were analysed on day 9 after the second IMRCs or IMRCs‐*MMP1 KO* transfusion (Figure 5I). Immunofluorescence analyses revealed that IMRCs directly degraded the pericellular collagen, while IMRCs‐*MMP1 KO* showed no such activity (Figure 5J,K). These findings demonstrate that IMRCs promote alveolar regeneration and suppress myofibroblasts by directly degrading collagen I via MMP1‐secreted protease activity in vivo.

### 3.6 IMRCs Transplantation Induces a Transitional Fibroblasts State Rather Than IMRCs‐ *MMP1 KO*

**FIGURE 6:**
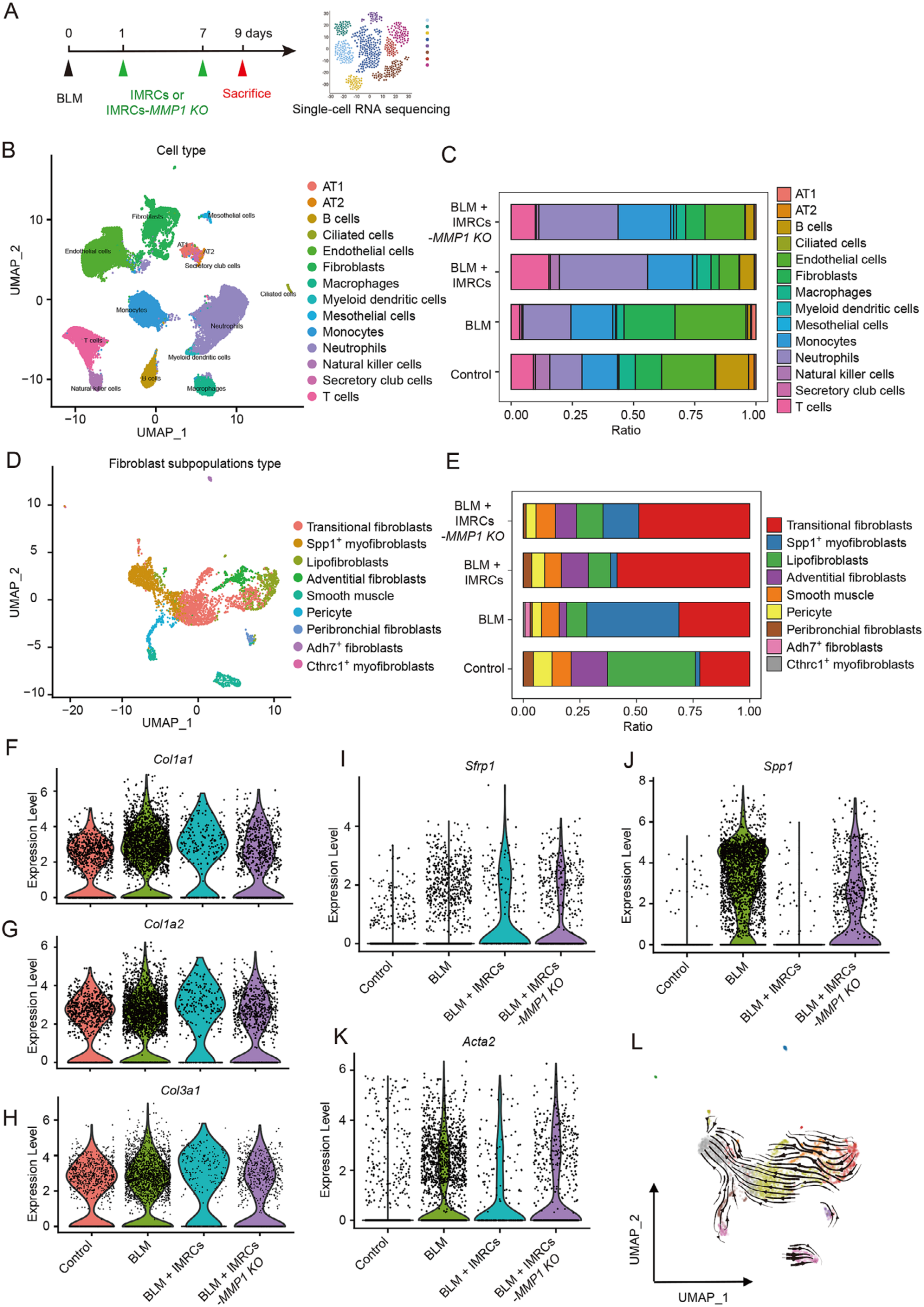
Heterogeneity of fibroblasts in the lung across treatments groups. (A) Experimental protocol schematic. For single‐cell RNA sequencing, lung tissues from *n* = 3 biologically independent mice per group were analysed. (B) Uniform manifold approximation and projection (UMAP) clustering depicts the cell types (38,534 cells) identified on day 9 after bleomycin injury. AT1, alveolar type I cells; AT2, alveolar type II cells. (C) Proportional distribution of cell types among treatments. (D) UMAP clustering depicts the fibroblast cell type (4275 cells). (E) Frequency distribution of fibroblast subpopulations across treatments. (F–K) Violin plots displaying fibroblast subtype‐specific gene expression across groups. (L) UMAP projection of single‐cell fibroblast populations overlaid with a vector field generated from pseudotime trajectory analysis. Arrows indicated the inferred directionality and flow of cellular state transition.

To investigate the impact of *MMP1* knockout on cellular distribution within lung tissue, we performed single‐cell transcriptome sequencing (scRNA‐seq) analysis for lung tissues collected on day 9 after bleomycin injury (Figure 6A). Uniform manifold approximation and projection (UMAP) analysis revealed 11 distinct cell clusters in mouse lungs: neutrophils, endothelial cells, monocytes, fibroblasts, T cells, B cells, macrophages, epithelial cells, natural killer cells, myeloid dendritic cells, and mesothelial cells (Figure 6B). Fibroblasts' distribution frequencies varied across treatment groups (Figure 6C). To further examine the effect of *MMP1* knockout on fibroblast activation, we performed subclustering analysis on Col1a1/Col1a2/Col3a1‐positive fibroblasts. Based on established methodologies [42], fibroblasts were classified into nine subpopulations: transitional fibroblasts, Spp1^+^ myofibroblasts, lipofibroblasts, adventitial fibroblasts, smooth muscle cells, pericytes, peribronchial fibroblasts, Adh7^+^ fibroblasts, and Cthrc1^+^ myofibroblasts (Figure 6D). Frequency distribution analysis revealed that in the BLM group, fibroblasts predominantly adopted the Spp1^+^ myofibroblasts phenotype, activated myofibroblasts. In contrast, IMRCs administration shifted fibroblasts toward a “transitional” state. *MMP1* knockout increased the proportion of Spp1^+^ myofibroblasts while reducing transitional fibroblasts (Figure 6E). Violin plots confirmed ubiquitous Col1a1/Col1a2/Col3a1 expression across all fibroblast subpopulations in every group (Figure 6F–H). The BLM group exhibited high expression of *Spp1* and *Acta2* (activated myofibroblasts' markers) but low *Sfrp1* expression (transitional fibroblasts' marker) (Figure 6I–K). Conversely, IMRCs transplantation induced high *Sfrp1* expression with low *Spp1* and *Acta2* levels, whereas *MMP1* knockout resulted in intermediate expression profiles of these genes (Figure 6I–K). To further elucidate the mechanism by which IMRCs suppress fibroblast activation beyond mere cell depletion, we performed pseudotime trajectory analysis on the single‐cell sequencing data (Figure 6L). The UMAP projection, overlaid with a vector field derived from pseudotime analysis, illustrated the dynamic transitions between fibroblast subpopulations. The arrows indicated the inferred directionality of cellular differentiation, revealing a prominent trajectory from transitional fibroblasts toward terminally activated Spp1^+^ myofibroblasts. We also performed a systematic analysis of intercellular communication strength among distinct cell subpopulations (Supporting Information, Figure S5). The results demonstrated that IMRCs attenuated the transmission of immune signals to transitional fibroblasts, which likely restricted pathological progression by effectively locking fibroblasts in an intermediate state and preventing their transition into Spp1^+^ myofibroblasts. Furthermore, IMRCs maintained stable communication between alveolar type I cells and fibroblasts and preserved the supportive signalling from lipofibroblasts or adventitial fibroblasts to alveolar type II cells, reflecting the preservation of alveolar homeostasis. Collectively, these findings suggested that MMP1 mitigated pulmonary fibrosis progression by maintaining physiological epithelial‐stromal homeostasis while suppressing immune cell‐driven terminal fibrosis. To elucidate the mechanism by which IMRCs regulate fibroblast phenotypes during pulmonary fibrosis and the potential confounding effects from alterations in cell apoptosis or proliferation, we performed TUNEL assays for apoptosis and Ki67 staining for proliferation in lung tissue sections. The results demonstrated that there were no significant differences in the percentage of TUNEL‐positive cells or Ki67‐positive cells among the groups (Supporting Information, Figure S4D–G). These findings indicated that the reduction of α‐SMA expression and the anti‐fibrotic effects observed in our study were not due to increased fibroblast apoptosis or inhibited proliferation. Instead, these results strongly support that IMRCs mediate fibroblast deactivation through a true phenotypic reversal, independent of altering cell survival or growth rates. These results indicated that IMRCs treatment promoted a transitional fibroblasts' state instead of activated myofibroblasts, while *MMP1* knockout altered IMRCs' modulatory effects on fibroblasts' activation progression.

## 4 Discussion

Our previous works have revealed that IMRCs robustly express *MMP1*, enabling them to reverse the upregulation of type I collagen induced by TGF‐β1 in vitro [27]. When administered to BLM‐induced PF models, IMRCs markedly reduced collagen deposition. As the key enzyme responsible for degrading fibrillar collagens (types I and III) in the extracellular matrix, MMP1 mediates critical pathways in both fibrosis development and resolution [43]. Numerous studies have confirmed that MMP1 promotes fibrosis reversal and functional tissue recovery in affected organs including lungs and liver [33, 44, 45]. In this study, we generated IMRCs‐*MMP1 KO* using CRISPR‐Cas9 technology, while also considering the possible off‐target effects. The sgRNA was designed using an online algorithm to minimize potential off‐target sites. Furthermore, the comprehensive characterization data presented in Figures 1 and 2 demonstrate that the IMRCs‐*MMP1 KO* cells retained all fundamental properties (morphology, proliferation, migration, surface marker profile, and immunomodulatory capacity) identical to wild‐type IMRCs. The high transcriptional similarity between IMRCs and IMRCs‐*MMP1 KO*, as revealed by RNA‐seq analysis, strongly suggests the absence of major unintended genomic alterations that would compromise core cellular functions. Therefore, the observed phenotypic differences are most likely attributable to the specific loss of MMP1.

In vitro, IMRCs‐*MMP1 KO* media failed to degrade deposited collagen I induced by TGF‐β1, unlike wild‐type IMRC media. In vivo, *MMP1* knockout significantly diminished the therapeutic efficacy of IMRCs against lung fibrosis. Subsequently, macrophage infiltration in lung tissue was detected. Analyses revealed equivalent reductions in macrophage infiltration with both IMRCs and IMRCs‐*MMP1 KO*. In vitro, IFN‐γ stimulation induced robust *IDO1* expression comparably in both cell types. Transcriptome profiling further confirmed equivalent anti‐inflammatory factor expression profiles between groups. These results indicated that *MMP1* deletion does not compromise the immunoregulatory functions characteristic of IMRCs. It is important to note that the bleomycin model features a dynamic immune response, where neutrophils are critical early drivers of injury and fibrosis initiation [46, 47]. Our data demonstrate that IMRCs' immunomodulatory secretome is intact without MMP1, suggesting that the immunoregulatory function is likely mediated by other factors via separate signalling pathways [39, 40, 41]. A logical and important future direction is to characterize the impact of IMRCs on the recruitment and function of other key immune cells, such as neutrophils and lymphocytes, across different phases of the fibrotic response. Elucidating these MMP1‐independent immunomodulatory pathways will provide a more complete understanding of how IMRCs shape the injury microenvironment and could inform their use in combination strategies targeting both inflammation and matrix deposition in progressive fibrotic diseases.

Strikingly, α‐SMA^+^ myofibroblast activity was significantly more suppressed in wild‐type IMRC‐treated mice versus IMRCs‐*MMP1 KO* recipients, indicating MMP1‐dependent inhibition of fibroblast activation. IMRCs‐*MMP1 KO* also impaired lung regeneration, evidenced by reduced pro‐SPC^+^ alveolar type II cell counts. Critically, collagen I deposition remained substantially elevated in IMRCs‐*MMP1 KO* recipients compared with wild‐type counterparts. Most significantly, in situ immunofluorescence demonstrated wild‐type IMRCs actively degrading pericellular collagen I fibres, whereas IMRCs‐*MMP1 KO* lacked this capability. These findings establish MMP1 secretion as the primary mechanism enabling IMRCs' direct collagenolytic activity. The pathogenesis of PF critically depends on the local tissue balance between MMPs and their tissue inhibitors (TIMPs). Following chronic lung injury, proliferating pulmonary myofibroblasts elevate TIMP‐1 expression while suppressing MMP1 activity. This MMP1/TIMP1 imbalance reduces degradation of type I and III collagens, driving pathogenic ECM accumulation that culminates in fibrosis [5]. Crucially, restoration of MMP1 expression has demonstrated fibrosis‐reversing capabilities across multiple studies. In liver fibrosis models, transplantation of MMP1‐overexpressing bone marrow‐derived MSCs (BM‐MSCs) significantly reduced collagen deposition while inhibiting hepatic stellate cell activation, resulting in substantial improvement of liver histopathology and function [33]. Most compellingly, direct administration of recombinant adenoviral MMP1 (Ad‐MMP1) in thioacetamide‐induced liver fibrosis models produced significant collagenolysis, reduced fibrotic burden, and restored hepatic function [44]. Collectively, these interventions confirm MMP1's therapeutic capacity to degrade structural collagens and reverse established fibrotic lesions.

Beyond chemical signalling, cells dynamically sense ECM physical properties including rigidity and viscoelasticity. Quantitative analysis confirms significantly increased ECM stiffness in IPF lungs compared with healthy tissue [48]. Pathologically stiff microenvironments directly promote lung fibroblast‐to‐myofibroblast transition, enhancing collagen secretion and TGF‐β pathway activation [49, 50]. When cultured on pathologically stiff substrates, lung fibroblasts undergo myofibroblast transdifferentiation, acquiring enhanced collagen production and TGF‐β signalling activity. In the early stage of liver fibrosis, hepatic sinusoidal endothelial cells can activate stellate cells (the effector cells of liver fibrosis) by transmitting mechanical signals through collagen fibre bundles [51]. Single‐cell mechanical stimulation, coupled with in vitro biomimetic models and mathematical modelling, has provided direct evidence for the interplay between fibroblasts and myofibroblasts, mediated by matrix fibre mechanical signals, which is named “paratensile signalling” [52]. In IPF, fibrotic ECM architecture mechanically constrains lung stem cell regeneration. MMP1‐mediated ECM degradation may reverse this restriction, potentially re‐establishing permissive microenvironments for lung stem cell expansion [53]. In our study, scRNA‐seq analysis showed that injured lungs primarily contain activated myofibroblasts (Spp1^+^ phenotype). Interestingly, IMRCs treatment induces a transitional fibroblast state to retard fibroblasts' activation. However, *MMP1* knockout alters IMRCs' modulatory effects on fibroblasts' activation progression. Consequently, IMRCs possess the potential to hinder the progression of fibrosis by secreting MMP1, which aids in degrading the extracellular matrix, thereby lessening ECM stiffness and impeding the transition of fibroblasts into myofibroblasts.

The ECM also serves as a reservoir for growth factors and cytokines, regulating their bioavailability and controlled release. Upon ECM degradation, sequestered growth factors and cytokines (e.g., VEGF, FGF2) are liberated, enhancing cell migration, proliferation, and differentiation—processes critical for tissue regeneration [54]. Studies confirm that MMPs can activate regenerative signalling pathways by releasing matrix‐bound growth factors through ECM proteolysis [31]. Specifically, MMP1 cleaves the IGF‐2/IGFBP2 complex, liberating IGF‐2 to activate IGF signalling pathways that enhance cellular migration [35]. MMP1 also proteolytically activates protease‐activated receptor 1 (PAR1) on cell surfaces, triggering intracellular G‐protein signalling cascades that potentiate cellular migration and invasion [36].

Although the role of MMP1 in the treatment of pulmonary fibrosis by IMRCs has been preliminarily explored, numerous challenges and issues still persist. While our data strongly support a mechanism centered on ECM remodelling and its biomechanical and biochemical consequences, future studies utilizing techniques such as proteomics or spatial transcriptomics on these defined fibroblast subpopulations will be invaluable to precisely delineate the downstream signalling pathways activated by MMP1‐mediated matrix degradation. While the present study definitively establishes the necessity of MMP1 for the anti‐fibrotic function of IMRCs through a clean genetic knockout approach, formal proof of sufficiency, such as rescuing the knockout phenotype by reconstituting MMP1 expression, represents an important future direction. Such experiments would require sophisticated design to mimic physiological secretion kinetics. Nonetheless, our finding that MMP1 is an essential functional mediator provides a strong rationale for its use as a key therapeutic biomarker in developing IMRC‐based therapies for IPF. In the preclinical studies, there is a pressing need for animal models that better mimic chronic IPF to advance our understanding of how IMRCs exert their therapeutic effects. Currently, BLM‐induced pulmonary fibrosis is still regarded as the most suitable animal model for preclinical evaluations [55, 56, 57]. Some studies show that in the early stage, stem cell transplantation effectively abrogates BLM‐induced lung injury by reducing inflammatory cell infiltration and promoting the repair of injured lungs [58, 59, 60]. However, increasing criticism suggests that potential therapies typically used within the first 7 days of exposure to BLM by preventing inflammation rather than reversing fibrosis may limit its applicability to human IPF [61]. We acknowledge that the classic intratracheal bleomycin mouse model, as employed in our study, induces a time‐dependent progression from acute inflammation to a peak of fibrosis around day 21, which may then partially resolve. Our administration timepoints (day 1 and day 7 post‐injury) were chosen to intervene during the active phase of injury response and early matrix deposition. The primary goal of this study was to dissect the specific functional mechanism by which IMRCs exert their therapeutic effect within a dynamic fibrotic setting. In clinical practice, most patients with interstitial lung disease often present with varying degrees of lung fibrosis. In this study, although the therapeutic effect of IMRCs in the early stage of BLM induced PF has been studied, the effect of IMRCs on the deposition of collagen remains elusive. Further investigation of IMRCs transplantation during advanced fibrotic stages is therefore essential to determine whether these cells can effectively eliminate established ECM deposits. Future mechanistic studies would benefit from implementing aged murine models and non‐human primate models of pulmonary fibrosis, which better recapitulate human disease progression and treatment responses.

## 5 Conclusion

In summary, the *MMP1* gene was successfully knockout without altering the fundamental characteristics or immune regulatory capabilities of IMRCs. However, *MMP1* knockout diminished the ability of IMRCs to degrade collagen I induced by TGF‐β1 in A549 cells. Notably, IMRCs demonstrated superior therapeutic efficacy and exhibited a remarkable capacity to degrade pericellular type I collagen and modulate fibroblast activation progression in fibrotic lung tissues in a MMP1‐dependent manner. Concisely, MMP1 plays a pivotal role and serves as a crucial therapeutic marker in the management of pulmonary fibrosis utilizing IMRCs.

## Author Contributions

Zhongwen Li, Dingyun Song, Faguo Sun and Licheng Song contributed equally to this original article. They completed the majority of the experiments, wrote the manuscript with help from all the authors. They also designed the experiments, performed the data curation and drafted the entire article. Bin An, Yao Tian, Tingting Gao, Jingjing Liu, Kan Zhang, Kaidi Liu, Kaiwei Wu, Yajing Li, Yi Yan, Yaru Liu, Ruofan Su, Zai Wang, Tiemei Zhao, Huaiyong Chen, Li Xiao, Liang Peng Liu Wang and Wei Li participated in the experiments and data analysis. Lixin Xie, Jie Hao, Huaping Dai and Jun Wu contributed to the conception, design of the study, the data analysis, the revision of the manuscript, and the final approval of the version to be published. All authors contributed to the article and approved the final version of the manuscript.

## Funding

This work was financially supported by the National Key Research and Development Program (2023YFC3605100 and 2020YFA0804000 to J.W., 2021YFA1101600 to L.W.), the National Natural Science Foundation of China (No. 32500705 to Z.L., No. 32370851 to J.W., No. 92068108 and No. 82370072 to H.D.), the Beijing Natural Science Foundation (Z230011 to W.L., Z240018 to J.W.), the Strategic Priority Research Program of the Chinese Academy of Sciences (XDC0200000 to W.L., XDA0510400 and XDC0250300 to J.W.), the CAS Project for Young Scientists in Basic Research (YSBR‐012 to W.L.), the Initiative Scientific Research Program, Institute of Zoology, Chinese Academy of Sciences (2023IOZ0101 to W.L.), the Chongqing Municipal Major Project for Technological Innovation and Applied Development (CSTB2025TIAD‐STX0044 to J.W.) and State Key Laboratory of Respiratory Health and Multimorbidity, State Key Laboratory Special Fund (2060204 to D.S.).

## Conflicts of Interest

The authors declare no conflicts of interest.

## Supporting information


**Figure S1:** Generation and characterization of *MMP1* knockout Immunity and Matrix‐Regulatory Cells (IMRCs‐*MMP1 KO*). (A) The sequencing chromatograms for at two independent clones. (B) Western blot for MMP1 protein expression in IMRCs and IMRCs‐*MMP1 KO*. (C) Quantification of the relative MMP1 protein expression levels in (B). Data are represented as the mean ± SEM, *n* = 3. ***p < 0.001. (D) The width of the scratch at 0, 24 and 48 h post‐scratch, comparing the healing progress of IMRCs and IMRCs‐*MMP1 KO* cultures. Data are represented as the mean ± SEM, *n* = 3.
**Figure S2:** *MMP1* knockout doesn't affect IMRCs' cytokines secretion characteristics. ELISA analysis of biologically relevant chemokines and cytokines in the conditioned medium of IMRCs and IMRCs‐*MMP1 KO* with or without IFN‐γ (100 ng/mL) stimulation. data are represented as the mean ± SEM, *n* = 3.
**Figure S3:** Knockout of *MMP1* affects the capacity of IMRCs to degrade collagen I induced by TGF‐β1 in vitro. (A) Diagram of the in vitro fibrosis protocol induced by 10 ng/mL TGF‐β1 with or without conditioned medium for 48 h in human lung fibroblasts (MRC‐5 cells). (B) Western blot for collagen I and α‐SMA protein expression in MRC‐5 cells with different treatment. (C) Quantification of the relative collagen I and α‐SMA protein expression levels in (B). Data are represented as the mean ± SEM, *n* = 3. *p < 0.05, **p < 0.01, ***p < 0.001, ****p < 0.0001.
**Figure S4:** IMRCs directly degrade collagen I through MMP1 secretion in vivo. (A) Western blot for Collagen I protein expression in mouse lung tissues with different treatment. (B) Quantification of the relative Collagen I protein expression levels in (A). Data are represented as the mean ± SEM, *n* = 3. (C) The hydroxyproline quantification assay on lung tissue homogenates from all experimental groups. Data are represented as the mean ± SEM, Control, *n* = 8; BLM, *n* = 8; BLM + IMRCs, *n* = 11 and BLM + IMRCs‐*MMP1 KO*, *n* = 9. *p < 0.05, **p < 0.01, ***p < 0.001. (D) Immunofluorescent analysis of α‐SMA and TUNEL protein expression in mice subjected to various interventions. Scale bars, 100 μm. (E) Immunofluorescent analysis of α‐SMA and Ki67 protein expression in mice subjected to various interventions. Scale bars, 100 μm. (F, G) Quantification of the TUNEL and Ki67 positive cells in (D and E). Data are represented as the mean ± SEM, Control, *n* = 8; BLM, *n* = 8; BLM + IMRCs, *n* = 11 and BLM + IMRCs‐*MMP1 KO*, *n* = 9.
**Figure S5:** The heatmap illustrated the intercellular communication intensity across distinct cell subpopulations. The *x*‐axis denotes signal receivers, while the y‐axis denotes signal senders. Red indicates stronger communication in IMRCs, whereas blue signifies stronger communication in the IMRCs‐*MMP1 KO* group.
**Table S1:** Primer sequences used in this study.
**Table S2:** Cell type specific marker genes.

**Table S3:** RNA sequencing gene expression profiles of all samples.

## Data Availability

All data generated or analysed during this study are included in this published article and its Supporting Information files.
