# Supplementary material for: Human Embryonic Stem Cell‐Derived Immunity‐And‐Matrix‐Regulatory Cells Attenuate Pulmonary Fibrosis via MMP1‐Mediated Collagen Degradation
Source: Cell Prolif. 2026 Aug 5:e70267. Online ahead of print. doi: 10.1111/cpr.70267 (PMC13439263; doi:10.1111/cpr.70267)
Supplement: Supplementary file 1 [file CPR-9999-e70267-s001.docx]

**Human embryonic stem cell-derived immunity-and-matrix-regulatory cells attenuate pulmonary fibrosis via MMP1-mediated collagen degradation**

**Materials and methods**

**Real-time quantitative PCR**

The total RNA was extracted using the RNAprep Pure Cell/Bacteria Kit (TIANGEN, Beijing, China; DP430) and subsequently reverse transcribed into cDNA using the PrimeScript™ First-Strand cDNA Synthesis Kit (TaKaRa, Shiga, Japan; 6110B). For quantitative analysis, real-time quantitative PCR (qPCR) was performed in the Light Cycler 480II system (Roche, Basel, Switzerland) employing SYBR Green Real-Time PCR Master Mix Plus (Toyobo, Osaka, Japan; QPS-201). GAPDH was used for internal normalization. The specific primers used for real-time PCR in this study are detailed in Supplementary information, Table S1.

**Enzyme-linked immunosorbent assay (ELISA)**

The levels of human MMP1 protein in the culture supernatant were measured by a Human MMP1 ELISA Kit (Cusabio, Wuhan, China; CSB-E04672h) according to the manufacturer’s instructions.

**Human MMP1 activity**

The IMRCs or IMRCs-*MMP1 KO* culture supernatants were collected to determine the activity of human MMP1 after 48 hours of culture. The activity of human MMP1 were measured by a SensoLyte® Plus 520 MMP-1 Assay Kit (AnaSpec, San Jose, CA, USA; AS-72012) according to the manufacturer’s instructions. Five-FAM/QXL®520 FRET peptide was used as MMP1 fluorogenic substrate. The fluorescence signal was monitored at Ex/Em = 490 nm / 520 nm upon MMP-1-induced cleavage of the FRET substrate.

**Growth curve**

IMRCs and IMRCs-*MMP1 KO* were seeded into 96-well plates at a density of 8 × 10^3^ cells per well. The cell growth was measured by Cell Counting Kit-8 (CCK8; Thermo Fisher Scientific, CK04). CCK8 solution was added on day 1, day 3, day 5 and day 7 by following the CCK8 kit manufacturer’s protocol. The absorbance was measured at 450 nm after 2 h of incubation.

**Flow cytometry**

Cells were harvested and blocked with 2% bovine serum albumin (BSA; Sigma-Aldrich, St. Louis, MO, USA; B2064) for 20 min at room temperature. Then, the cells were stained with fluorescein-conjugated antibodies for 40 min at room temperature in 1% BSA. After incubation, cells were washed 3 times with phosphate buffered saline (PBS) and analyzed with MoFlo (Beckman, Brea, CA, USA) and associated software. The antibodies used for flow cytometry were as follows: PE-conjugated mouse anti-human CD105 (Biolegend, San Diego, CA, USA; 323206), PE-conjugated mouse anti-human CD90 (eBioscience, San Diego, CA, USA; 12-0909-42), PE-conjugated mouse anti-human CD29 (Biolegend, 303004), PE-conjugated mouse anti-human CD73 (BD Biosciences, San Jose, CA, USA; 550257), PE-conjugated mouse anti-human CD45 (BD Biosciences, 560957), PE-conjugated mouse anti-human CD34 (BD Biosciences, 555822), PE-conjugated mouse anti-human HLA-ABC (BD Biosciences, 560965), PE-conjugated mouse anti-human HLA-DR (BD Biosciences, 555561), and the PE-conjugated mouse IgG1 (BD Biosciences, 551436) as an isotype control.

**Scratch assay for cell migration**

The wound gap was generated using the Ibidi Culture-Inserts (Ibidi, Martin Reid, Germany; 80209). IMRCs and IMRCs-*MMP1 KO* single cell suspensions were seeded into Culture-Inserts at a density of 1 × 10^5^ cells / mL. For each group, three repeated holes were set in parallel. Once the cells had adhered, the Culture-Inserts were removed. At 0 h, 24 h and 48 h after incubation, the width of the scratch was observed.

**RNA-seq library preparation and data analysis**

Total RNA was isolated from cells by Trizol (Invitrogen, Waltham, MA, USA; 15596018). The NEBNext^®^Ultra^TM^ RNA Library Prep Kit for Illumina^®^ was used to prepare RNA-seq libraries. Sequencing was performed on an Illumina HiSeq X-Ten sequencer (Illumina, San Diego, California, USA) with 150 bp paired-end sequencing reaction. All RNA-sequencing data analysis was performed with Hisat2 (version 2.1.0) and Cufflinks (version 2.2.1) using the UCSC hg19 annotation with default settings. Only reads mapping to unique genome locations and genes with a minimum of 1 FPKM in at least one sample were considered for further analysis. Differentially expressed genes (DEGs) were determined using a two-fold change threshold, and genes showing less than two-fold increased or decreased expression were not considered significant. Clustering and heatmap analysis were performed utilizing the hierarchical clustering functionality and the heatmap.2 function from the R statistical computing language.

**Cells culture**

A549 and MRC-5 cells were purchased from Beina Biology (Shanghai, China) and cultured in Ham’s F-12K medium (Gibco, Carlsbad, MO, USA; 21127022) with 10% fetal bovine serum (FBS; Gibco, 10091-148). A549 were treated with 10 ng/mL TGF-β1 (R&D systems, MN, USA; 240-B) to induce epithelial-mesenchymal transition. MRC-5 were treated with 10 ng/mL TGF-β1 to induce fibroblast activation.

**Cytokine Analysis**

Conditioned medium of IMRCs, IMRCs-*MMP1 KO* or with IFN-γ (100 ng/mL) stimulation were collected. Forty-eight cytokines including FGF basic, Eotaxin (also known as CCL11), G-CSF (CSF3), GMCSF (CSF2), IFN-γ, IL-1β, IL-1ra, IL-2, IL-3, IL-4, IL-5, IL-6, IL- 7, IL-8 (also known as CXCL8), IL-9, IL-10, IL-12 (p70), IL-12 (p40), IL-13, IL-15, IL-17A, IP10 (CXCL10), MCP-1 (CCL2), MIP-1a(CCL3), MIP-1b (CCL4), PDGF-BB, RANTES (CCL5), TNF-α, VEGF, IL-1α, IL-2Ra, IL-16, GRO-α, HGF, IFN-α2, LIF, MCP-3, MIG, B-NGF, SCF, SCGF-β, SDF-1α, CTACK, MIF, TRAIL, IL-18, M-CSF, TNF-β were detected by using the premixed 48-plex Bio-Plex Pro Human Cytokine Assay (Bio-Rad, Hercules, CA, USA; 12007283) according to the manufacturer’s instructions.

**Immunofluorescence staining**

Cells were fixed with 4% paraformaldehyde (PFA) (Aladdin Chemical Co., Ltd., C104190) for 15 min. The cells were washed 3 times with PBS and then permeabilized with 0.5% Triton X-100 for 15 min and blocked with 2% BSA for 60 min at room temperature. Then, the cells were stained with 1:200 mouse monoclonal IgG E-cadherin (Abcam, San Francisco, CA, USA; ab1416) and 1:200 rabbit monoclonal IgG Collagen I (Cell Signaling Technology, Danvers, MA, USA; 84336) overnight at 4°C in 2% BSA. The cells were washed 3 times with PBS and then incubated with 1:200 Alexa Fluor 488 donkey anti-mouse IgG (H+L) (Jackson ImmunoResearch, West Grove, PA, USA; 715-545-151) or 1:200 Fluorescein (Cy3) donkey anti-rabbit IgG (H+L) (Jackson ImmunoResearch, 711-165-152) secondary antibodies in 2% BSA for 60 min at room temperature in the dark. The washing step was repeated before staining the nuclei with Hoechst 33342 (Invitrogen, H3570).

**Lung function**

The lung function was detected by flexiVent FX experimental platform (SCIREQ, Montreal, Canada) according to the manufacturer’s instructions. On day 21, mice administered with 3% pentobarbital sodium (40 mg/kg) intraperitoneally were endotracheally intubated in the middle of a tracheotomy. The indicators of lung function including pressure-volume (PV) curve, forced vital capacity (FVC), static compliance (Crs), inspiratory capacity (IC), respiratory resistance (Rrs), elastic resistance (Ers), tissue damping (G) and tissue elasticity (H) were obtained.

**Lung coefficient**

The lung tissue was weighed and the lung coefficient was calculated according to the formula: lung coefficient = lung mass (g) / body mass (kg).

**Micro-CT**

Mice were anesthetized with isoflurane (RWD, Shenzhen, China; R510-22-10) for micro-CT scans on day 14 and 21 after BLM injury. Mice micro-CT scans were performed in Quantum FX system (PerkinElmer, Waltham, MA, USA) according to the manufacturer’s instructions.

**Histological staining and immunofluorescence staining analysis**

The lung tissue was dehydrated, paraffin-embedded, and cut into 4 µm sections. These lung sections were then stained with Hematoxylin and Eosin (H&E) as well as Masson's trichrome stain to comprehensively evaluate pathological changes. The Szapiel score was used to quantify alveolitis and the Ashcroft score was used to quantify pulmonary lesions. Immunofluorescence staining was performed using antibodies against α-smooth muscle actin (α-SMA; Servicebio, Wuhan, China; GB13044), collagen I (Servicebio, GB13091), F4/80 (Servicebio, GB11027), Ki67 (ServiceBio, GB111141), TUNEL (ServiceBio, G1501), proSPC (Millipore, Burlington, MA; AB3786) and human HLA-ABC (Abcam, ab225636). ImageJ software was used to analyze histochemistry and fluorescence images. For Ashcroft and Szapiel scoring, optical microscopy (Carl Zeiss, Oberkohen, Germany; Axioscope5) was employed at 100× and 200× magnifications. From each of the six randomly selected sections, three areas were randomly chosen for analysis. The scores were independently evaluated by two experimenters to ensure accuracy and consistency.

**Western blot**

Cells and lung tissues were harvested in RIPA Lysis Buffer (strong) (CWbio, Taizhou, China; CW2333S) containing protease inhibitors (Roche, 4693124001). A total of 40 µg proteins were separated by 4%–20% gradient SDS-PAGE gels (GenScript, Nanjing, China; M42015C) and transferred to PVDF membranes (Millipore, Billerica, MA, USA; IPVH00010). The membranes were blocked at room temperature with 1% BSA for 1 h and incubated overnight at 4°C with primary antibodies: mouse MMP1 antibody (Cell Signaling Technology, 84336), rabbit Collagen I antibody (Cell Signaling Technology, 84336), rabbit α-SMA antibody (Sigma-Aldrich, A5228) and rabbit β-actin antibody (Sigma-Aldrich, SAB5600204). Then, the membranes were washed with TBST for 3 times and incubated for 1 h with a secondary antibody: anti-mouse IgG antibody (HRD) (Sigma- Aldrich, A9044) and anti-rabbit IgG antibody (HRD) (Sigma-Aldrich, A0545) at room temperature. Images were obtained using the ChemiDoc XRS + imaging system (Bio-Rad) and quantified using the Quantity One software.

**Analysis of collagen by hydroxyproline quantification**

Pre-cooled PBS (0.01 M, pH 7.4) is used to rinse, which is then weighed and minced. The minced tissue is combined with PBS (containing protease inhibitors) at a 1:9 weight-to-volume ratio and transferred into a glass homogenizer. The mixture is thoroughly homogenized on ice. Finally, the homogenate is centrifuged at 5000 × g for 5 minutes, and the supernatant is collected for analysis. The levels of mouse collagen I protein were measured by a mouse hydroxyproline ELISA Kit (BYabscience, Nanjing, China; BY-WJZF1269) according to the manufacturer’s instructions.

**Figure. S1** **Generation and characterization of *MMP1* knockout Immunity- and Matrix-Regulatory Cells (IMRCs-*MMP1 KO*). (A)** The sequencing chromatograms for at two independent clones. **(B)** Western blot for MMP1 protein expression in IMRCs and IMRCs-*MMP1 KO*. **(C)** Quantification of the relative MMP1 protein expression levels in **(B)**. Data are represented as the mean ± SEM, n=3. ****p* < 0.001. **(D)** The width of the scratch at 0, 24 and 48 h post-scratch, comparing the healing progress of IMRCs and IMRCs-*MMP1 KO* cultures. Data are represented as the mean ± SEM, n=3.

**Figure. S2 *MMP1* knockout doesn’t affect IMRCs’ cytokines secretion characteristics.** ELISA analysis of biologically relevant chemokines and cytokines in the conditioned medium of IMRCs and IMRCs-*MMP1 KO* with or without IFN-γ (100 ng/mL) stimulation. data are represented as the mean ± SEM, n=3.

**Figure. S3 Knockout of *MMP1* affects the capacity of IMRCs to degrade collagen I induced by TGF-β1 *in vitro*.** (**A)** Diagram of the *in vitro* fibrosis protocol induced by 10 ng/mL TGF-β1 with or without conditioned medium for 48 h in human lung fibroblasts (MRC-5 cells). **(B)** Western blot for collagen I and α-SMA protein expression in MRC-5 cells with different treatment. (**C**) Quantification of the relative collagen I and α-SMA protein expression levels in (**B**). Data are represented as the mean ± SEM, n=3. **p* < 0.05, ***p* < 0.01, ****p* < 0.001, *****p* < 0.0001.

**Figure. S4 IMRCs directly degrade collagen I through MMP1 secretion *in vivo*. (A)** Western blot for Collagen I protein expression in mouse lung tissues with different treatment. **(B)** Quantification of the relative Collagen I protein expression levels in **(A)**. Data are represented as the mean ± SEM, n=3. **(C)** The hydroxyproline quantification assay on lung tissue homogenates from all experimental groups. Data are represented as the mean ± SEM, Control, n=8; BLM, n=8; BLM + IMRCs, n=11 and BLM + IMRCs-*MMP1 KO*, n=9. **p* < 0.05, ***p* < 0.01, ****p* < 0.001. **(D)** Immunofluorescent analysis of α-SMA and TUNEL protein expression in mice subjected to various interventions. Scale bars, 100 µm. **(E)** Immunofluorescent analysis of α-SMA and Ki67 protein expression in mice subjected to various interventions. Scale bars, 100 µm. **(F, G)** Quantification of the TUNEL and Ki67 positive cells in (**D** and **E**). Data are represented as the mean ± SEM, Control, n=8; BLM, n=8; BLM + IMRCs, n=11 and BLM + IMRCs-*MMP1 KO*, n=9.

**Figure. S5 The heatmap illustrated the intercellular communication intensity across distinct cell subpopulations.** The x-axis denotes signal receivers, while the y-axis denotes signal senders. Red indicates stronger communication in IMRCs, whereas blue signifies stronger communication in the IMRCs-*MMP1 KO* group.

**Supplementary Table S1** Primer sequences used in this study.

| Gene name | Forward (5^’^-3’) | Reverse (5’-3’) |
| --- | --- | --- |
| *MMP1* | TGCTTCCCTGAGACCCAGTT | GATCACTTCTTTCCTTTGCATCAAG |
| *IDO1* | GCCAGCTTCGAGAAAGAGTTG | ATCCCAGAACTAGACGTGCAA |
| *CDH1* | AAAGGCCCATTTCCTAAAAACCT | TGCGTTCTCTATCCAGAGGCT |
| *ACTA2* | AAAAGACAGCTACGTGGGTGA | GCCATGTTCTATCGGGTACTTC |
| *COLLAGEN I* | GAGGGCCAAGACGAAGACATC | CAGATCACGTCATCGCACAAC |
| *TGFβ1* | CTAATGGTGGAAACCCACAACG | TATCGCCAGGAATTGTTGCTG |
| *GAPDH* | CTCTGCTCCTCCTGTTCGAC | CGACCAAATCCGTTGACTCC |

**Supplementary Table S2** Cell type specific marker genes.

| Cell types | Genes |
| --- | --- |
| Alveolar type I cells | *AGER, PDPN, CAV1, HOPX, AQP5* |
| Alveolar type II cells | *SFTPC, SFTPA1, ABCA3, LPCAT1* |
| Neutrophils | *G0s2, Fcgr3* |
| Endothelial cells | *Cldn5, Pecam1* |
| Monocytes | *Fcna, Cd14* |
| Fibroblasts | *Col1a1, Col1a2, Col3a1* |
| T cell | *Cd3d, Cd3e, Cd3g* |
| B cell | *Cd79a, Mzb1, Ms4a1, Cd79b* |
| Macrophages | *Marco, Msr1, Mrc1, Itgax* |
| Natural killer cells | *Klrk1, Klrd1* |
| Myeloid dendritic cells | *Cd83, Cd86* |
| Mesothelial cells | *Wt1, Upk3b, Msln, Col8a1* |
| Secretory club cells | *SCGB1A1* |
| Ciliated cells | *Foxj1, Rsph1, Cdhr3, Cdhr4* |
